# Infection with *Burkholderia pseudomallei* – immune correlates of survival in acute melioidosis

**DOI:** 10.1038/s41598-017-12331-5

**Published:** 2017-09-22

**Authors:** Susanna J. Dunachie, Kemajittra Jenjaroen, Catherine J. Reynolds, Kathryn J. Quigley, Ruhena Sergeant, Manutsanun Sumonwiriya, Panjaporn Chaichana, Suchintana Chumseng, Pitchayanant Ariyaprasert, Patricia Lassaux, Louise Gourlay, Charuporn Promwong, Prapit Teparrukkul, Direk Limmathurotsakul, Nicholas P. J. Day, Daniel M. Altmann, Rosemary J. Boyton

**Affiliations:** 1 Mahidol-Oxford Tropical Medicine Research Unit, Mahidol University, Bangkok, Thailand; 2 Centre for Tropical Medicine and Global Health, University of Oxford, Oxford, United Kingdom; 3 Department of Medicine, Imperial College London, London, United Kingdom; 4 Department of Biosciences, University of Milan, Milan, Italy; 5 Sunpasitthiprasong Hospital, Ubon Ratchathani, Thailand; 6 Department of Tropical Hygiene, Faculty of Tropical Medicine, Mahidol University, Bangkok, Thailand

## Abstract

Melioidosis, caused by *Burkholderia pseudomallei*, is a potentially lethal infection with no licensed vaccine. There is little understanding of why some exposed individuals have no symptoms, while others rapidly progress to sepsis and death, or why diabetes confers increased susceptibility. We prospectively recruited a cohort of 183 acute melioidosis patients and 21 control subjects from Northeast Thailand and studied immune parameters in the context of survival status and the presence or absence of diabetes. HLA-B*46 (one of the commonest HLA class I alleles in SE Asia) and HLA-C*01 were associated with an increased risk of death (odds ratio 2.8 and 3.1 respectively). Transcriptomic analysis during acute infection in diabetics indicated the importance of interplay between immune pathways including those involved in antigen presentation, chemotaxis, innate and adaptive immunity and their regulation. Survival was associated with enhanced T cell immunity to nine of fifteen immunodominant antigens analysed including AhpC (BPSL2096), BopE (BPSS1525), PilO (BPSS1599), ATP binding protein (BPSS1385) and an uncharacterised protein (BPSL2520). T cell immunity to GroEL (BPSL2697) was specifically impaired in diabetic individuals. This characterization of immunity associated with survival during acute infection offers insights into correlates of protection and a foundation for design of an effective multivalent vaccine.

## Introduction

Melioidosis is a disease caused by the Gram-negative soil-dwelling bacterium *Burkholderia pseudomallei* (Bp). A major cause of mortality in Southeast Asia and Northern Australia, it is now recognised as a significant cause of disease in tropical regions worldwide^1,2^. In-hospital mortality in Northeast Thailand is 40%^3^. Using a model that mapped cases and environmental Bp, the estimated global burden of melioidosis is 165,000 human melioidosis cases/pa, causing 89,000 deaths^2^. Furthermore, the model predicts melioidosis under-reporting. Key risk factors are diabetes mellitus, alcohol consumption, chronic renal and lung disease, and increasing age^4^. A vaccine targeting at risk groups is an urgent unmet need.

Studies in patients and mouse models have sought to identify immune correlates of protection from Bp, but there is a lack of consensus about the qualities required of protective vaccine candidates. Bp is an intracellular pathogen^5^, so an effective vaccine is likely to require induction of humoral and cellular immunity. T cell immunity to several Bp antigens has been described in exposed individuals^6–8^ and survivors of acute melioidosis^9^.

Mapping the immunomics of Bp exposure poses significant challenges: the Bp genome is over seven megabase pairs in size and contains more than 5,000 coding sequences^10^. Felgner and colleagues used a protein array platform to screen 1,205 proteins^11^ against 747 individual human sera and identified forty-nine immunoreactive Bp proteins. Based on data indicating that these forty-nine proteins contained both B cell and T cell epitopes, work within the NIH/NIAID-funded Immune Epitope Database (IEDB) consortium then sought to develop this into a systematic approach for Bp T cell antigen discovery, predominantly in HLA transgenic mice and in asymptomatic, seropositive human donors^12–14^. Of the 49 candidate, T cells antigens, a short-list of 15 was here selected for study, based on ease of synthesis and solubility, and prior experience in terms of their ability to elicit T cell responses. Thus, in the present study, the short-listed, 15 Bp proteins were chosen for screening of T cell immunogenicity by interferon-gamma (IFNγ) ELISpots in patient cohorts from northeast Thailand with culture-confirmed acute melioidosis, and again twelve weeks after recovery, alongside endemic controls seronegative for Bp by indirect haemagglutination assay (IHA). Results were analysed by survival and diabetic status. HLA-B*46, and HLA-C*01, were associated with increased risk of death. T cell immunity to GroEL (BPSL2697) was specifically impaired in diabetic individuals and transcriptomic analysis during acute infection in diabetics demonstrated the importance of immune pathways involved in antigen presentation, chemotaxis, innate and adaptive immunity and their regulation. We identified five protein antigens that are immunodominant in survivors: BPSS1525 (BopE) – a Type III secreted protein, BPSL2096 (AhpC) – the alkyl hydroperoxide reductase, BPSL2520 – an uncharacterised secreted protein, BPSS1385 (ATP binding protein) – a Type III effector protein, and BPSS1599 (PilO) – a Type IV pilus biosynthesis protein. T cell responses to these antigens offer insights into correlates of protection making them attractive candidates for development of a vaccine platform.

## Results

### Survival following acute melioidosis is associated with enhanced T cell responses to immunodominant Bp antigens

Bp-specific T cell immunity was assessed in 44 melioidosis patients from Ubon Ratchathani, a melioidosis endemic region of Thailand, compared to 21 local, seronegative healthy controls identified from blood donors at Sunpasitthiprasong Hospital. Of the 44 melioidosis patients, 13 died within 28 days of admission to hospital. Peripheral blood was collected during the acute illness and again from survivors at 12 weeks. Diabetic status at the time of diagnosis of melioidosis was recorded.

**Figure 1:**
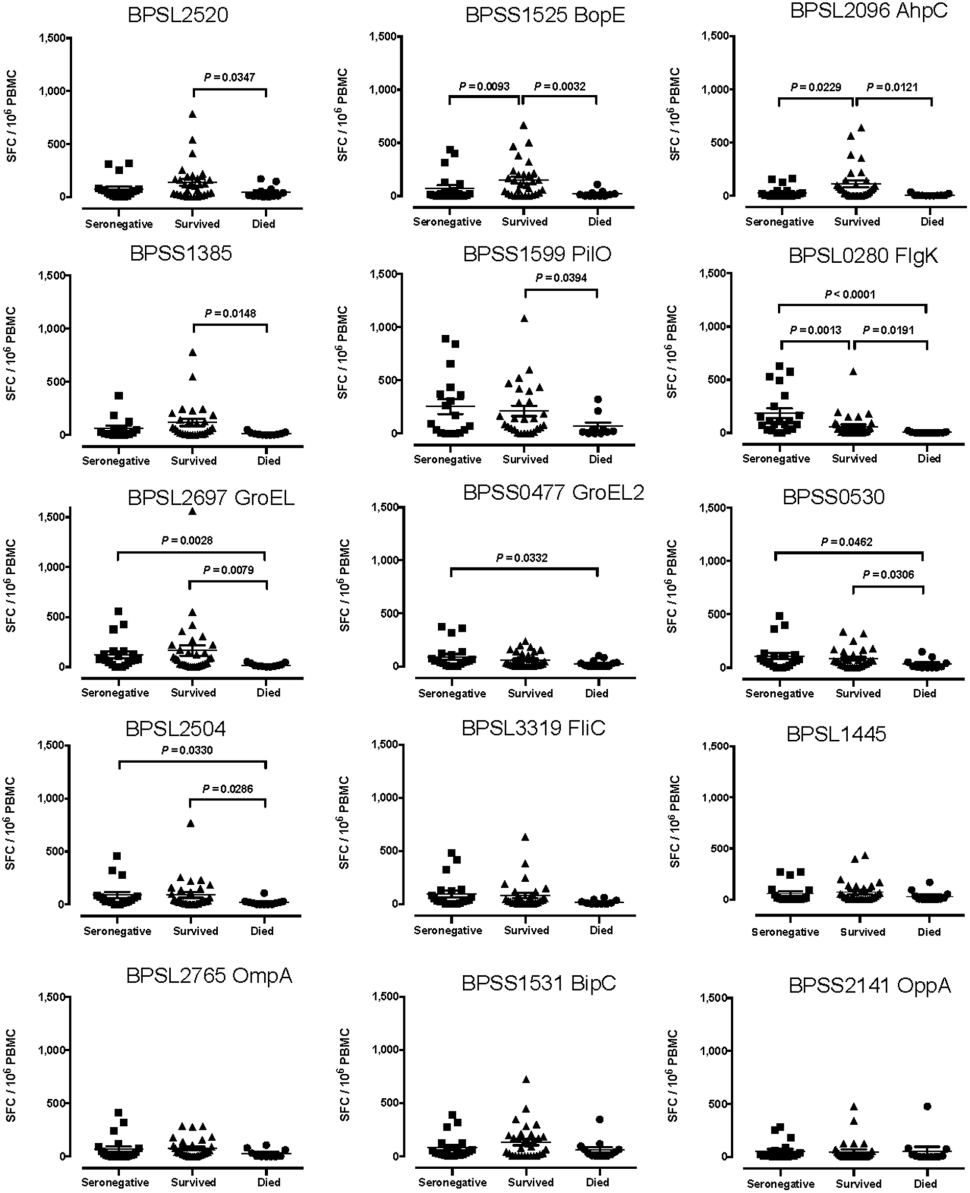
Enhanced adaptive immunity to 9 *Burkholderia pseudomallei* antigens during acute melioidosis in survivors compared to fatal cases. IFNγ ELISpot assays for HIA-Bp or one of 15 Bp antigens were performed on cryopreserved PBMC samples from 18 seronegative controls (black squares), 30 acute melioidosis survivors (black triangles) and 12 fatal cases (black circles). Data shown for each antigen is the number of spot forming cells (SFC) per 10^6^ PBMC following subtraction of the number of SFC from culture with media alone. Error bars represent mean + SEM and statistical significance between groups was determined using the Mann Whitney test.

**Figure 2:**
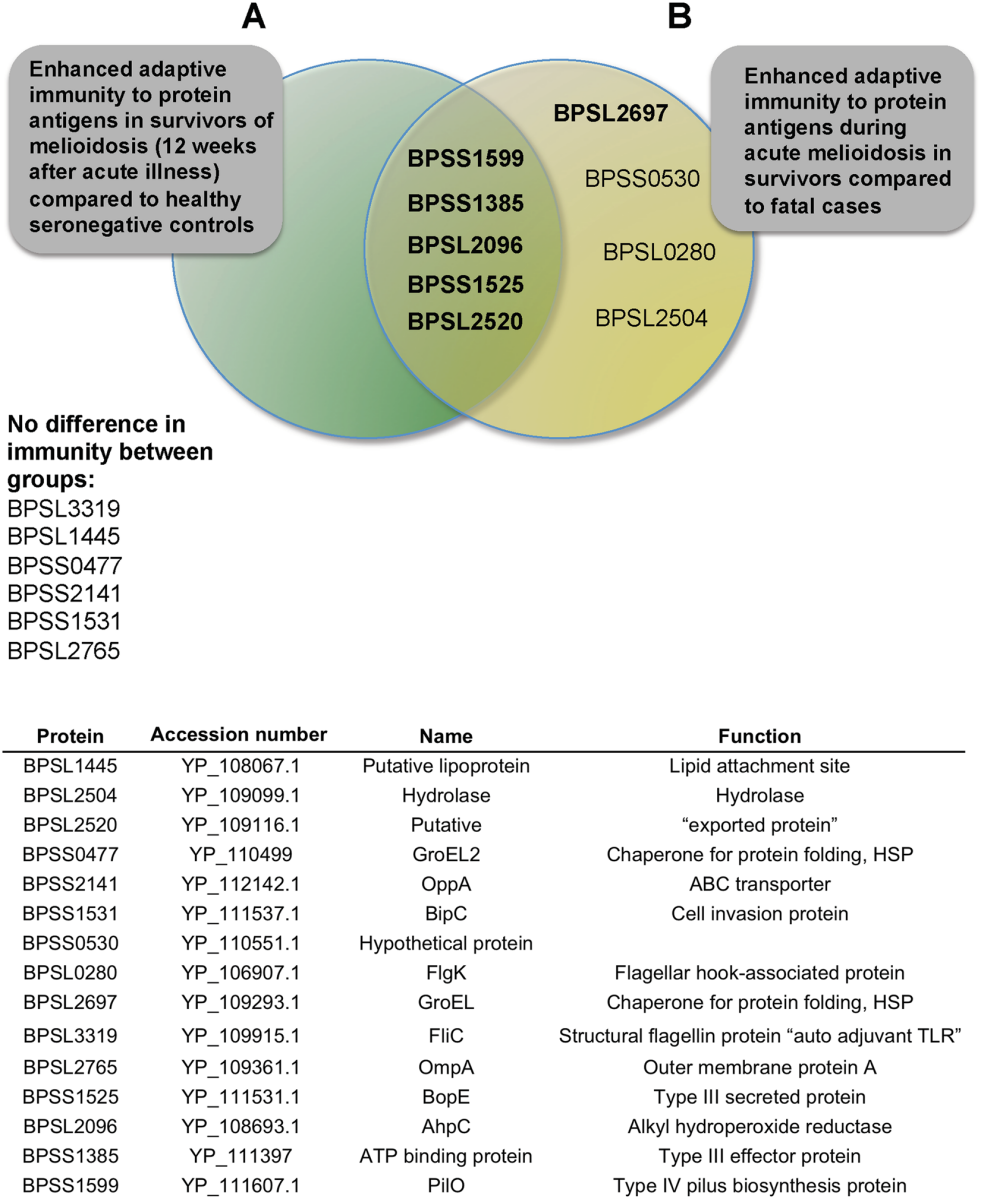
Enhanced adaptive immunity to Bp antigens are seen in melioidosis survivors, both at the time of acute infection and 12 weeks after recovery from acute infection. The Venn diagram shows the overlap between enhanced T cell responses at the time of acute melioidosis in survivors compared to fatal cases (9 Bp antigens) and at 12 weeks after recovery in survivors compared to seronegative controls (5 Bp antigens). T cell responses to 6 Bp proteins showed no difference between the patient groups tested (**A**). The table shows the *B. pseudomallei* strain K96243 protein antigens selected for investigation (**B**).

We studied T cell correlates of disease outcome by comparing T cell immunity in patients who died with that of survivors. Bp protein antigen-specific T cell responses at the time of acute infection were lower in patients who died. In particular, T cell responses to 9 of the 15 antigens studied (BPSL2520, BPSS1525, BPSL2096, BPSS1385, BPSS1599, BPSL0280, BPSL2697, BPSS0530, BPSL2504) were significantly lower (Figs 1 and 2). One of these, BPSL2096 (AhpC) has previously been reported by us as a strong antigen across diverse HLA types^14^.

Some seronegative controls showed T cell responses to Bp antigens (Fig. 1), possibly reflecting cross-reactivity with environmental species such as *B. thailandensis*. For example, BPSS1599 (PilO) responses were raised in controls; there is 91% homology between PilO of *pseudomallei* and *thailandensis* (http://www.mgc.ac.cn/cgi-bin/VFs/compvfs.cgi?Genus=Burkholderia). Comparisons were made with healthy control subjects from the region with antibody titers by IHA assay below 1:40. However, not everyone exposed to *Bp* has a positive IHA titre. An Australian longitudinal study of culture-confirmed cases of melioidosis, reported 13% of subjects seronegative (IHA < 1:40)^15^.

**Figure 3:**
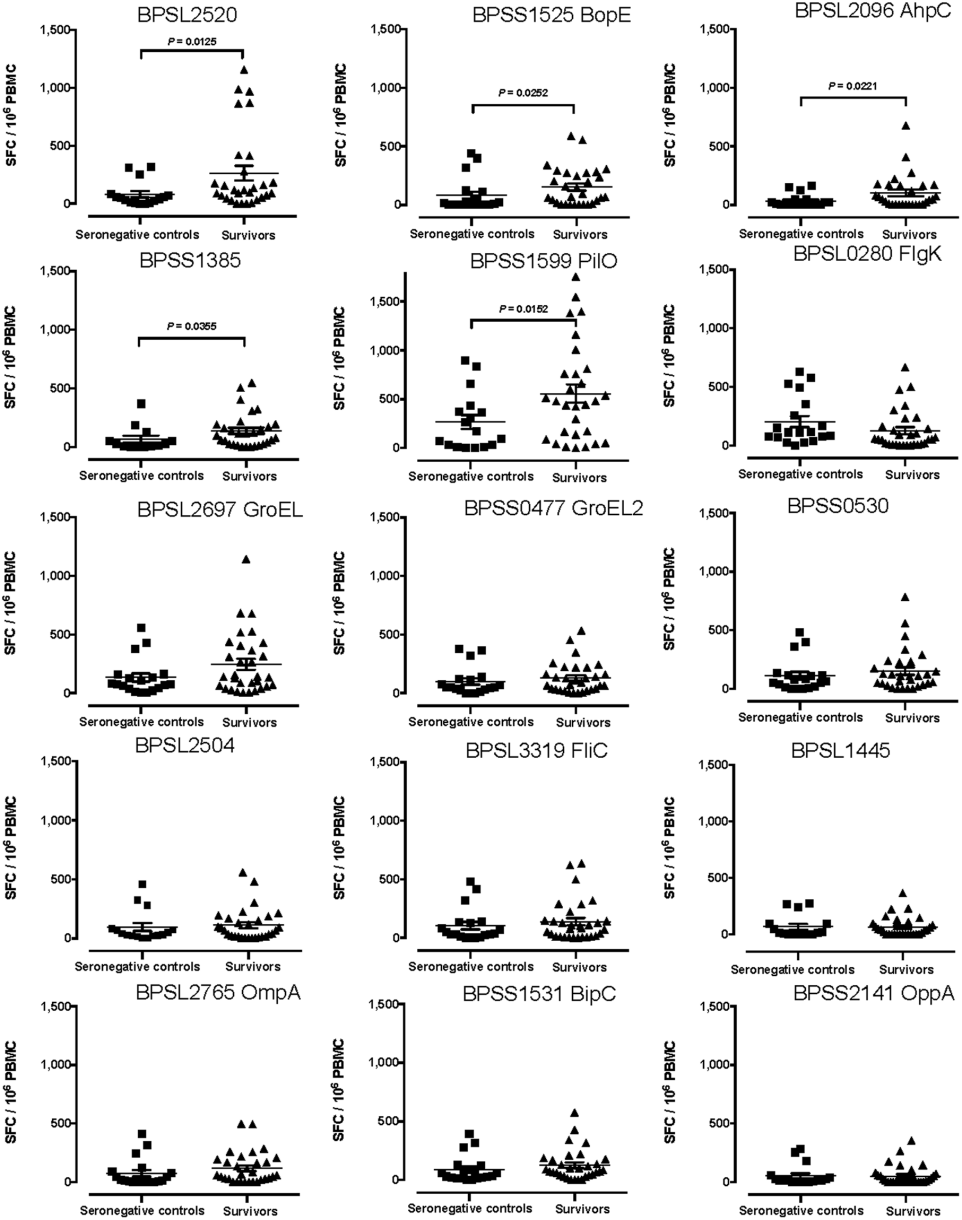
Enhanced adaptive immunity to 5 *Burkholderia pseudomallei* antigens in survivors of melioidosis, 12 weeks after acute illness, compared to healthy seronegative controls. IFNγ ELISpot assays for HIA-Bp or one of 15 Bp proteins were performed on cryopreserved PBMC from 16 seronegative controls (black squares) and 30 acute melioidosis survivors (black triangles). PBMC samples from melioidosis survivors were taken 12 weeks after acute illness. Data shown for each protein antigen is the number of spot forming cells (SFC) per 10^6^ PBMC following subtraction of the number of SFC from culture with media alone. Error bars represent mean + SEM and statistical significance between groups was determined using the Mann Whitney test.

We then analysed T cell responses to Bp protein antigens in patients who survived infection, 12 weeks after admission with acute melioidosis. Five of fifteen Bp antigens tested showed significantly greater responses in convalescent melioidosis patients compared to seronegative controls (Figs 2 and 3). The five protein antigens identified as immunodominant in survivors were: BPSS1525 (Type III secreted protein, BopE), BPSL2096 (alkyl hydroperoxide reductase, AhpC), BPSL2520 (uncharacterised secreted protein), BPSS1385 (type III effector protein, ATP binding protein), and BPSS1599 (Type IV pilus biosynthesis protein, PilO). This is in line with the notion that a large and diverse T cell repertoire controls infection.

**Figure 4:**
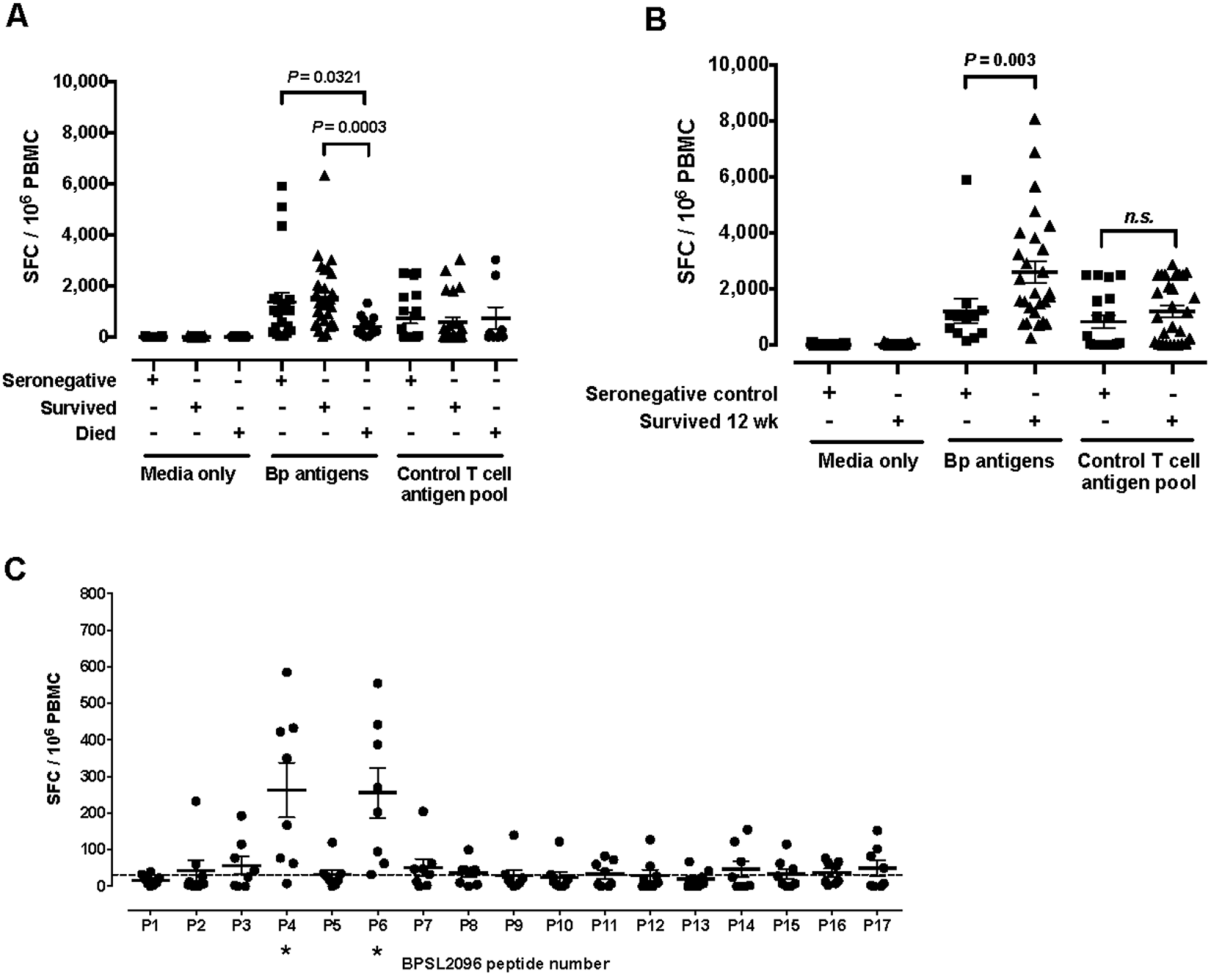
Magnitude of T cell responses to Bp antigens in survivors and fatal cases compared to T cell immunity to Bp antigen BPSL2096 is concentrated on 2 peptide epitopes. IFNγ T cell responses to all 15 Bp proteins and to a control T cell antigen pool were summed for each individual in the seronegative control (black squares, n = 18) melioidosis survivor (black triangles, n = 30) and melioidosis fatality (black circles, n = 12) groups. Data are shown for both acute melioidosis (**A**) and at 12 weeks post acute illness (**B**). Error bars represent mean + SEM and statistical significance between groups was determined using the Mann Whitney test. IFNγ ELISpot assays were performed for a BPSL2096 20mer peptide panel. A positive response (*) was defined as 2 SD above the mean of the media only control and is represented by a dotted line. Error bars represent mean + SEM (**C**).

Enhanced immunity 12 weeks after recovery from acute infection is shown in the cumulative antigen responses to all fifteen Bp antigens (Fig. 4). T cell responses at the time of acute infection are higher in survivors than those who died (Fig. 4a), and in recovered individuals 12 weeks after acute infection than in seronegative controls (Fig. 4b). The median, summed IFNу ELIspot response for survivors is 1,301 SFC/10^6^ compared to 234 SFC/10^6^ for fatal cases (p = 0.0003). This is the result of a defect in specific immunity to Bp rather than a wider breakdown of immunity resulting from acute sepsis, since no significant difference was seen for T cell responses to the CEF peptide pool. While some individuals fail to show a response to the CEF peptide pool, it will be noted that in Fig. 4b this non-responsiveness is equally distributed between patients and controls, and that a much smaller number of patients show baseline responses to Bp antigens than to the CEF peptide pool. At 12 weeks there is a higher cumulative frequency of Bp-reactive T cells in convalescent patients (Fig. 4b) than during acute melioidosis.

While these IFNγ ELIspot assays were designed primarily to identify CD4 T cell epitopes (exogenous supply antigen-presenting cells [APC] with 20mer peptides that, in the absence of uptake and processing into the class I pathway, are too large for an HLA class I groove) we have previously described Bp antigens presented via class I and II^14^. CD8 T cell responses to Bp are of interest, this being an intracellular pathogen of APCs where antigen presentation by access to the class I pathway is required for CD8 recognition. We therefore examined the source of the antigen-specific IFNγ signal in responding cells by intracellular cytokine staining (ICS) gating on CD4 or CD8 (Supplementary Fig. 1). Responses were predominantly through CD4 T cells for BPSL2096 AhpC, but either CD4 or CD8 T cells for two of the other antigens tested (BPSL2520 and BPSS1525 (BopE). T cell responses to individual peptide epitopes of BPSL2096 (AhpC) in survivors of melioidosis were evaluated by *ex-vivo* IFNγ ELIspot assay using 20-mer peptides overlapping by 10 amino acids covering the sequence of BPSL2096 (AhpC) (Supplementary Table 3). Two immunodominant peptides were identified, peptides 4 and 6 of BPSL2096 (AhpC) (Fig. 4c). Peptide 6 from BPSL2096 (AhpC) has previously been reported by us as a strong peptide antigen that can be presented across diverse HLA types^14^.

Thus, we have identified T cell mediated correlates of protection during acute infection with melioidosis and 12 weeks after recovery.

### Antibody recognition of BPSL2096 (AhpC) is a marker of acute melioidosis, while strong antibody responses to BPSS1525 (BopE) are associated with survival

**Figure 5:**
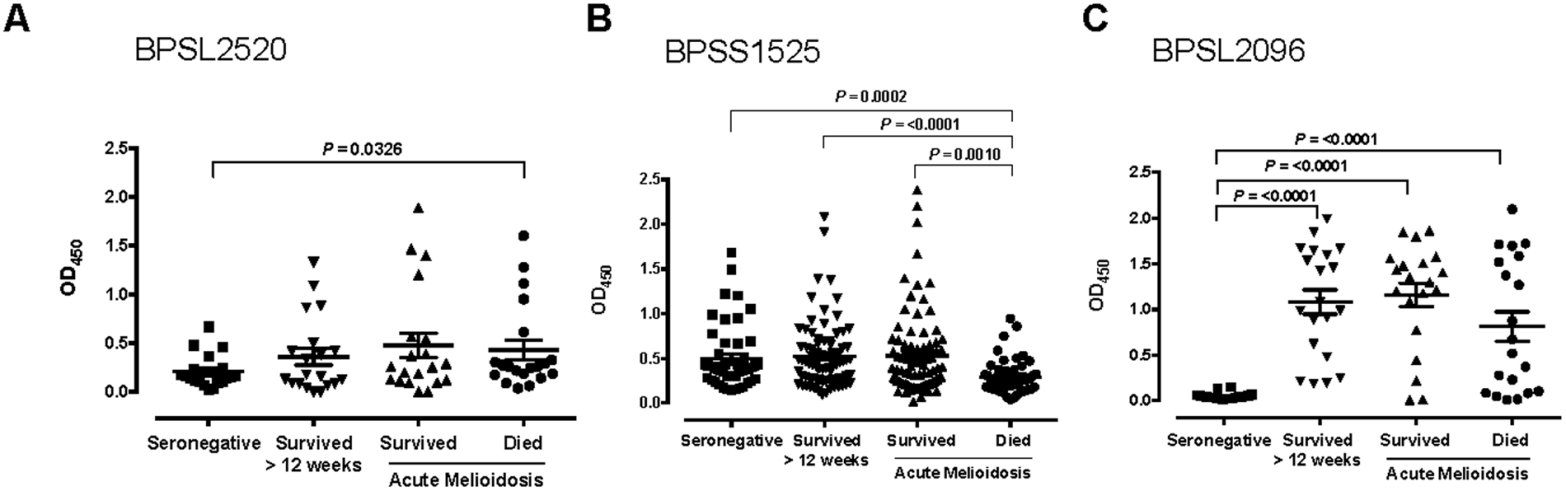
Fatal cases of acute melioidosis show reduced antibody responses to Bp antigen BPSS1525 compared to seronegative controls. Serum samples from seronegative controls (black squares), acute melioidosis survivors (upward black triangles), acute melioidosis fatal cases (black circles) and melioidosis survivors 12 weeks after acute illness (downward black triangles) (n = 20 per group) were used to measure antibody titres against the Bp antigens BPSL2520 **(A)**, BPSS1525 **(B)** and BPSL2096 **(C)**. Error bars represent mean + SEM and statistical significance between groups was determined using the Mann Whitney test.

Three of the protein antigens that elicited the strongest T cell responses in survivors, BPSL2520, BPSS1525 (BopE) and BPSL2096 (AhpC), were tested for antibody seroreactivity (Fig. 5a,b,c). Antibody levels were measured for 21 seronegative controls, for 83 surviving melioidosis patients during acute disease and in convalescence at 12 weeks, and for 47 acute melioidosis patients who died. Antibody responses to BPSS1525 (BopE) are associated with survival (p = 0.001) from acute infection, mirroring the differential T cell response to this antigen (Fig. 5b). Strong positive antibody responses to BPSL2096 (AhpC) are associated with Bp infection and a history of melioidosis (Fig. 5c).

### The presence of class I alleles HLA-B*46, and HLA-C*01 are associated with increased mortality following acute melioidosis

In line with a possible role of CD8 (and/or NK cell) immunity, we found an increased risk for death from melioidosis in individuals carrying the class I alleles, HLA-B * 46 or HLA-C * 01 (Supplementary Tables 1 and 2). For individuals carrying an HLA- B * 46 allele, the odds ratio (OR) for death from acute melioidosis was 2.8 (95% confidence interval CI 1.3–5.7, p = 0.005). For individuals carrying an HLA- C * 01 allele, the OR for death from acute melioidosis was 3.1 (95% CI 1.5–6.2, p = 0.002).

### Acute melioidosis in the presence of concurrent diabetes is associated with a reduced T cell response to the GroEL (BPSL2697) and GroEL2 (BPSS0477) stress proteins

**Figure 6:**
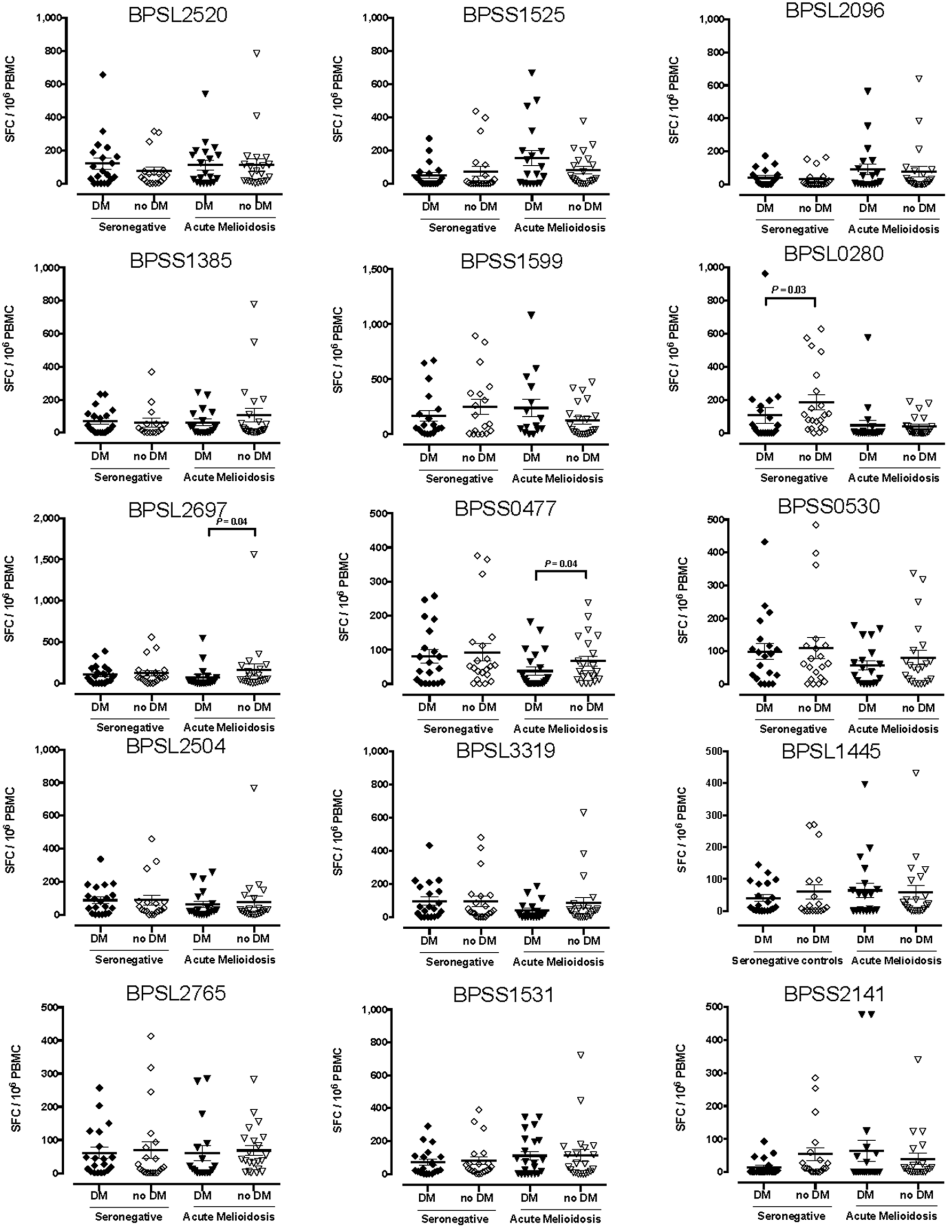
Diabetic patients with acute melioidosis show a reduced T cell responses to heat shock proteins GroEL and GroEL2 compared to non-diabetic melioidosis patients. IFNγ ELISpot assays for HIA-Bp and one of 15 Bp antigens were performed on cryopreserved PBMC samples from 20 diabetic seronegative controls (black diamonds), 18 non-diabetic seronegative controls (open diamonds), 20 diabetic acute melioidosis patients (black triangles) and 22 non-diabetic acute melioidosis patients (open triangles). Data shown for each antigen is the number of spot forming cells (SFC) per 10^6^ PBMC following subtraction of the number of SFC from culture with media alone. Error bars represent mean + SEM and statistical significance between groups was determined using the Mann Whitney test.

Diabetes is a risk factor for melioidosis, although the immune mechanisms underlying susceptibility are not fully understood^16^. We, therefore, explored functional immune differences between individuals with and without diabetes. Half of the acute melioidosis patients in this cohort had a concurrent diagnosis of diabetes. Diabetic individuals had reduced T cell immunity to two GroEL protein antigens, BPSL2697, GroEL, and BPSS0477, GroEL2 during acute infection compared to non-diabetics (Fig. 6). Altered T cell immunity to the GroEL antigens is of interest, since cross-reactivity between microbial GroEL antigens and mammalian Hsp60 homologues has been implicated in the immunopathogenesis of diabetes^17,18^. It is hypothetically possible that responsiveness to Bp GroEL antigens was reduced through regulation or exhaustion of responsiveness to the endogenous homologue. To interrogate this premise further, transgenic mice expressing the human HLA-DR1 heterodimer were immunized with recombinant BPSL2697 (GroEL) and T cell peptide epitopes mapped by analyzing recall to a peptide panel comprising BPSL2679 (GroEL) peptides 20aa in length overlapping by 10aa (Supplementary Table 4). Four HLA-DR1 restricted peptide epitopes were identified: p15, p18, p22 and p30 (Supplementary Fig. 2a). HLA-DR1 transgenic mice were then immunized with recombinant BPSL2697 (GroEL) and T cell recall responses analysed against the peptide library for bacterial BPSL2697 (GroEL) or the homologous sequence from human Hsp60 (Supplementary Table 5). There was no evidence to support immunological cross-reactivity at the T cell level in this experiment (Supplementary Fig. 2b,c).

### Acute melioidosis in the presence of concurrent diabetes is associated with suppressed immune activation

Diabetic individuals with acute melioidosis show differential and suppressed immune activation compared to those without diabetes. PBMC from 24 patients with acute melioidosis showed greater cytokine release of IL-10 and IL-23 from PBMC of non-diabetic compared to diabetic individuals. There were no differences for IL-17A, IL-4, and IL-13 (Supplementary Fig. 3).

### Host immunity to Bp in acute melioidosis in diabetic individuals is associated with distinctive transcriptomic changes

To identify further the transcriptomic changes associated with host immunity to Bp, we cultured PBMC from acutely infected diabetic patients for 18 hours in the presence of either heat-killed Bp (HkBp) or one of three Bp antigens (BPSL2520, BPSS1525, or BPSL2096). Since the presence of diabetes is the major comorbidity associated with increased susceptibility to melioidosis (Supplementary Table 6), our initial focus was to characterise the nature of immune responsiveness during acute infection, within this group. Comparison with non-diabetic donors was not undertaken at this stage due to the confounder of the diverse array of other comorbidities, making this a phenotypically heterogeneous group to study.

**Figure 7:**
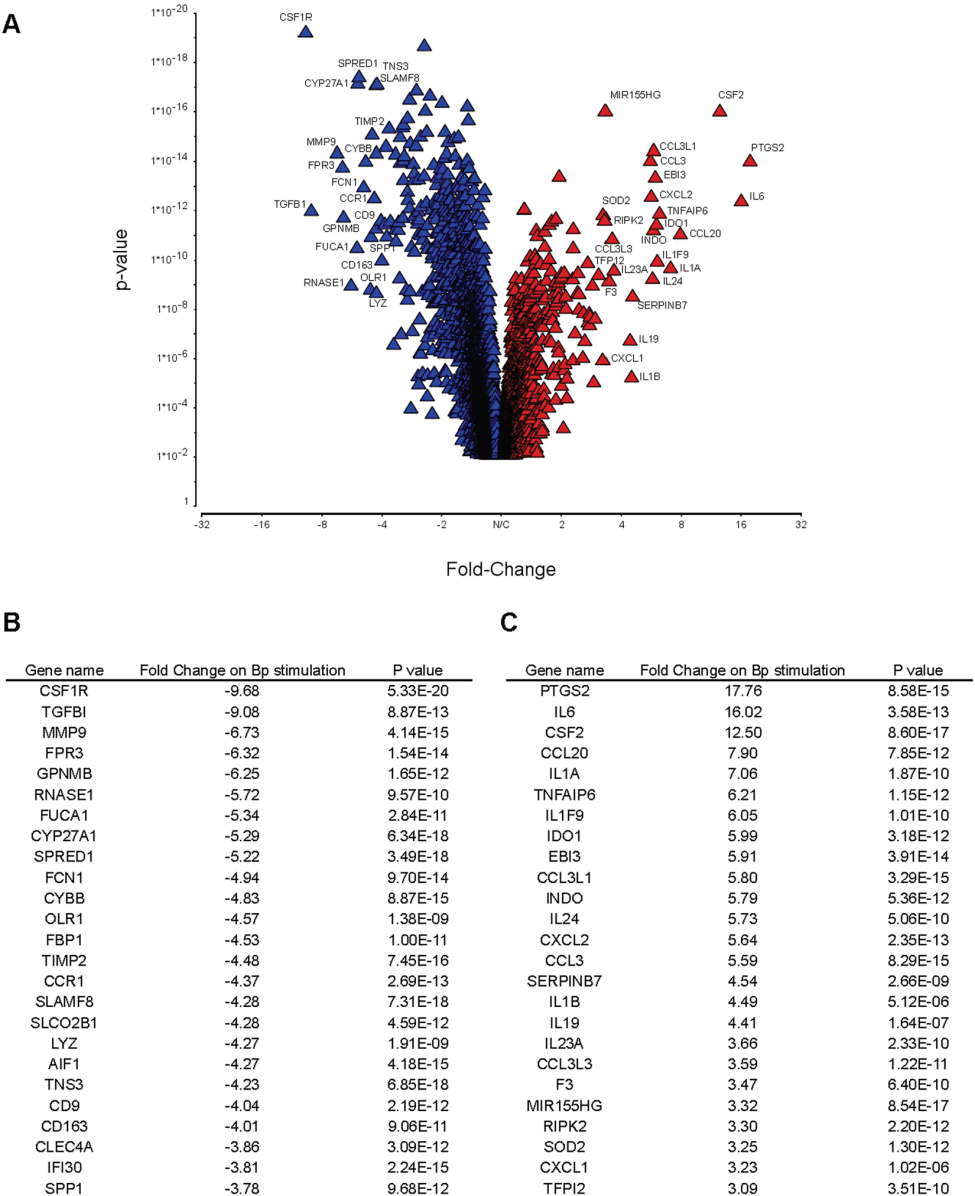
Transcriptomic analysis during acute infection in diabetics demonstrates altered regulation of a wide variety of immune related genes following stimulation of PBMC with HIA-Bp. PBMC from acute melioidosis patients were stimulated with either HIA-Bp or media alone for 18 h before extraction of RNA and transcriptomic analysis using an Illumina bead array. Genes significantly up or down-regulated following HIA-Bp stimulation compared to media alone were determined using a paired ANOVA, FDR adjusted with a P value cut off of 0.05. Statistically significant genes are represented in a volcano plot (**A**) with the top 25 downregulated (red triangle) and 25 upregulated (blue triangle) genes annotated (**B**,**C**).

Several of the transcripts up-regulated in response to HkBp were those that might be expected to contribute either to initial innate inflammation or to arming of adaptive immunity and chemotaxis (Fig. 7a,b,c and Supplementary Tables 7 and 8). Within this group are PTGS2/COX2, CSF2, IL-6, IL-1A, IL-23A and CCL20. Up-regulation of MiR-155 is of interest since it is implicated in pro- and anti-inflammatory settings, notably in this context as the regulator of a network that actively orchestrates macrophage survival and T cell immunity in Mtb infection^19^. The down-regulated transcripts are more functionally diverse, including CSF1R, TGFB1, MMP9 and FPR3.

A total of 1,751 genes were differentially expressed in response to each of all three of the Bp antigens tested separately. Pathways identified with high enrichment scores and significant P values included lysosome and phagosome pathways, cytokine and cytokine receptor pathways, Toll-like receptor signalling pathways, and antigen processing and presentation pathways. Notably, there was up-regulation of components of the MHC class I and down regulation of components of MHC class II antigen processing and presentation pathways.

## Discussion

Bp is associated with diverse clinical outcomes ranging from asymptomatic seroconversion, to localised infection, sepsis and death. Of around 5,000 proteins encoded in the genome, several hundred are seroreactive. A multi-antigen vaccine platform inducing protective humoral and cellular immunity against Bp likely needs to overcome redundancy in bacterial virulence factors and host evasion strategies. It is important to look beyond reactogenicity and seek host immunity that contributes to protection, as for Salmonella^20^; hence there is urgent need to characterise those antigen responses involved in protection from a lethal outcome, and generally to define immune correlates of clinical outcome. Target populations for any melioidosis vaccine include individuals with defined risk factors such as diabetes mellitus, making an understanding of the immune deficits in diabetic individuals that predispose to susceptibility to this bacterium extremely important.

Here, we show enhanced T cell immunity to nine specific antigens in individuals who survived the acute infection compared to those who died. Five of these antigens (PilO, ATP binding, AhpC, BopE and the uncharacterised BPSL2520) elicited a memory response in the survivors 12 weeks after acute infection (compared to healthy seronegative controls).

Based on studies in HLA transgenic mice, we have previously highlighted BPSL2096 (AhpC) as a highly immunogenic Bp antigen^14^. Across several intracellular bacterial pathogens, the alkyl hydroperoxide reductases are up-regulated in response to host oxidative stress, yet this up-regulation exposes them as prominent targets for adaptive immunity. A 40-fold up-regulation of AhpC has been implicated in resistance to high concentrations of reactive oxygen species by *B. cenocepacia* strains from biofilms, making it a virulence factor for Burkholderia^21^. AhpC was highly immunogenic for T cells both in our cohort and in a separate cohort of exposed individuals in Khon Kaen, northeast Thailand^14^. Enhanced T cell immunity to AhpC was observed in survivors compared to fatal cases during acute melioidosis, and in survivors compared to healthy seronegative controls. The pattern of immunodominant T cell epitopes from AhpC was similar to epitope mapping results in HLA-DR and -DQ transgenics^14^, with p6 immunodominant in human populations and in HLA transgenics. While the case for host-dependent selection pressure is a difficult one to make for Bp, it is noteworthy that p6, a T cell epitope highlighted in the present study, as in our earlier work, was found to have been deleted from a pathogenic, clinical isolate from Cambodia^14^. Although the Bp immunome is clearly large and complex, the definition of highly immunodominant, common, peptide-HLA complexes offers the potential for developments in future immune monitoring of immune status^12–14,22,23^. A new tool developed from analysis of Bp pMHC and *Pseudomonas aerguniosa* ^24^ pMHC complexes in responder datasets is BIITE, a means of elucidating likely pMHC combinations out of bulk PBMC responses in HLA-heterozygous individuals who will be expressing multiple HLA class II heterodimers to present peptide^23^. AhpC, and specifically, the p6 epitope from this antigen, stand out as a part of the Bp immunome that is highly visible to the T cell repertoire.

While those individuals who succumbed to fatal infection showed almost complete shutdown of specific T cell responses to Bp antigens, this was pathogen-specific rather than a function of more general immune shutdown in sepsis^25^, with no differential impairment in responses to control antigens. Thus, while this case is generally argued in terms of systemic immune susceptibility, partly through Treg function, our findings offer an alternate scenario whereby there is a specifically impaired response to Bp antigens, more compatible with the notion of tolerogenic presentation to effector T cells. Individuals with diabetes have altered innate and adaptive immunity to infection, with differences described in several cellular processes including neutrophil, macrophage, NK and lymphocyte function^16^. There is considerable healthcare concern about the ‘double burden’ of increased risk from the convergence of non communicable diseases (NCDs) and infection: in this case the prevalence of diabetes increasing in Asia in countries which also carry a burden of intracellular bacterial infections such as Burkholderia and tuberculosis^26^. In this cohort, half of acute melioidosis patients had concurrent diabetes. Diabetes in Northern Thailand has not been extensively characterized, but is typically adult onset, not initially requiring insulin, and frequently occurring in individuals of normal BMI. Improved understanding of the enhanced susceptibility to intracellular infections in diabetics is urgently needed. Here, diabetic individuals showed lower T cell immunity to GroEL proteins during acute melioidosis compared to non-diabetic patients. GroEL proteins, which have been explored as vaccine candidates in Shigella^27^ are molecular chaperones involved in folding of newly synthesised proteins and maintenance of protein homeostasis^28^. The eukaryote protein Hsp60 is structurally and functionally highly similar to GroEL. Presumably as a consequence of this homology, autoimmunity to self-Hsp60 following infection can be a feature of autoimmune diseases, including diabetes^29^; it is interesting in this context that we observed specifically down-regulated responses to Bp GroEL proteins in diabetic individuals. This may indicate immune regulation or exhaustion of this repertoire as a consequence of diabetogenesis, leaving a deficit also in the anti-bacterial response. T cell responses to heat-killed Bp are suppressed in diabetics with acute melioidosis compared to non-diabetics^9^, and lower responses to GroEL could be part of this mechanism.

Our study showed reduced IL-23 and IL-10 cytokine responses in diabetic individuals, suggesting dysregulated bacterial immunity. Cytokine production by macrophages in response to infection has previously been reported as lower in diabetic mice^30^ and diabetic people^31^, with reduced macrophage migration and phagocytosis^32^. We found decreased responses in healthy diabetics compared to healthy non-diabetic controls to another flagellar subunit protein, BPSL0280 FlgK (Fig. 5), the flagellar hook-associated protein. Antibody responses to FlgK have been noted as significantly raised in recovered melioidosis patients; FlgK is intrinsically cytotoxic to macrophages, though it remains to be seen whether this might facilitate or impair subsequent FlgK presentation^33^.

HLA genotyping of this acute melioidosis cohort identified increased mortality associated with the presence of HLA-B46 and HLA-C * 01. HLA-B46 and HLA-C * 01 are in linkage disequilibrium. Both of these alleles could function in this context either to present peptide to CD8 T cells or to interact with inhibitory killer immunoglobulin-like receptors (KIR) of NK cells. The presence of these alleles is associated with a lethal outcome, suggesting highly impaired host immunity. HLA-B46 is one of the most common HLA-B alleles in SE Asia. However, it is associated with several negative disease outcomes, including enhanced risk of cerebral malaria^34^. It has been proposed that HLA-B46 has a novel peptide binding groove, presenting a highly constrained peptide pool^35^.

Transcriptomic studies of murine infection have been reported^36^, and an analysis of unstimulated whole blood from patients with acute melioidosis has previously demonstrated a gene expression signature dominated by interferon-signalling pathways similar to tuberculosis infection^37^. Our analysis considered Bp specific immunity during acute melioidosis in diabetics. There is a strong picture of early integration and up-regulation of innate and adaptive immune pathways. This includes innate cytokines such as IL-1A, TNFA1 and IL-6, as well as cytokines polarising adaptive Th17 immunity such as IL-23A. Bp infection of primary human monocytes has been shown to activate gene expression of IL-23^38,39^. This is somewhat different from a recent report of cytokine transcription in unstimulated PBMC of melioidosis cases, which emphasised global up-regulation of Th17 and Th2 pathways^39^. Interestingly, transcripts associated with regulation of adaptive immunity, such as IDO-1 and IL-24, are activated. Several key changes relate to chemotaxis or mobilisation of appropriate subsets – CSF2, CCL20, CXCL2 and CCL3. Further studies will be required to interrogate the specific functional implications of these pathways for acute immune responses to Bp. The strong up-regulation of the immunomodulatory microRNA, MiR-155, is reminiscent of observations in *M. tuberculosis* infection of macrophages^19^. In that setting a dual activity of MiR-155 was proposed, both maintaining the survival of infected macrophages and promoting survival of responding T cells. It is also noteworthy that PTGS2/COX2 was so strongly up-regulated, since two studies have shown impaired host defence against Bp through COX2/PGE2^40,41^. Our new transcriptomic findings offer a mechanistic rationale for the efficacy of COX-2 inhibitors, tolfenamic acid (TA) and NS398, in the post-exposure protection of Bp challenged mice, and support further efforts to develop clinical therapeutics targeting the COX2 pathway.

In summary, we have characterised immune correlates of protection in a cohort of acute melioidosis patients. HLA-B46 and HLA-C*01, were associated with increased mortality. We identified antigens and epitopes, immunodominant in survivors compared to endemic controls: BPSS1525 (BopE) – a Type III secreted protein, BPSL2096 (AhpC) - the alkyl hydroperoxide reductase, BPSL2520 – an uncharacterised secreted protein, BPSS1385 (ATP binding protein) a Type III effector protein, and BPSS1599 (PilO) – a Type IV pilus biosynthesis protein and peptides 4 and 6 from BPSL2096 (AhpC). Antibody responses to BPSL2096 (AhpC) were associated with a history of acute melioidosis, while strong antibody responses to BPSS1525 (BopE) were associated with survival. Impaired responses to GroEL proteins were demonstrated in diabetics during acute infection. Transcriptomic analysis of acute infection in diabetics illuminates the interplay of pathways controlling chemotaxis, antigen presentation, innate and adaptive immunity, as well as their regulation. Overall, differential host immunity to these antigens offers insights into correlates of protection, making them attractive candidates for development of a vaccine platform.

## Materials and Methods

### Ethics statement

Mouse experiments were performed in accordance with UK Home Office legislation under the terms of Home Office Project License Project License PPL 70/7708, Imperial College London UK granted for this work under the “Animals (Scientific Procedures) Act 1986”. Local ethical review and formal approval was also obtained through the Imperial College Ethical Review Process Committee.

Human studies and consent forms were approved by the ethics committees of Faculty of Tropical Medicine, Mahidol University, of Sunpasitthiprasong Hospital, Ubon Ratchathani and the Oxford Tropical Research Ethics Committee. The study was conducted according to the principles of the Declaration of Helsinki (2008) and the International Conference on Harmonization (ICH) Good Clinical Practice (GCP) guidelines. Written informed consent was obtained for all patients enrolled in the study.

### HLA genotyping

Genomic DNA was isolated from peripheral blood by high salt extraction. HLA‐A, B, and C loci were typed by SSOP PCR.

### Subjects

For human immunology studies, blood samples were collected from 183 patients over 18 years of age with cultured-confirmed melioidosis at Sunpasitthiprasong Hospital, Ubon Ratchathani at a median of 5 days after admission to hospital (IQR 4–6 days), and from 83 of these patients after recovery 12 weeks later (Supplementary Table 1). 44/183 patients (24%) died within 28 days. This is in line with a larger published cohort from the same hospital^9^. Healthy control subjects were recruited from the blood donation clinic at Sunpasitthiprasong Hospital, and 21 subjects were selected as seronegative controls for melioidosis if their indirect hemagglutination assay (IHA) titre was <1:40^15,42^. Of the 183 patients with a diagnosis of acute melioidosis studied on enrolment (week 0), 123 subjects (67%) had a diagnosis of diabetes (defined for the purpose of this study as a past medical history of diabetes and / or a blood glycated haemoglobin (HbA_1c_) of ≥7%. For patients who did not attend follow-up, their 28-day survival status was determined using hospital mortality records and contact by telephone. Here, we analyse immune correlates of host immunity to Bp in a total cohort of 189 acute melioidosis patients, 44 of whom died (Table S1). Immune correlates of mortality were characterised at the level of HLA class I alleles (n = 189), T cell IFNγ response to fifteen immunodominant Bp antigens (n = 44), T cell epitope mapping (n = 26), and antibody responses (n = 130). Cytokine response profiles (n = 44) and comprehensive transcriptomics of antigen-stimulated PBMC from infected diabetic individuals (n = 13) were also studied.

### Antigens and peptides

Fifteen antigens from *B. pseudomallei* strain K96243 were selected for investigation (Fig. 2). Protein sequences were codon optimised, sub-cloned into an *E-Coli* expression vector, and purified using a His-Tag. Proteins were checked by SDS-PAGE and western blot, using an anti 6-His antibody. All proteins were produced by Biomatik (Biomatik USA, Delaware, USA). BPSS1599 was produced as previously described (Lassaux *et al*. 2014 PMID: 24728008). Unless otherwise stated, Bp antigens were used individually in all assays at a final concentration of 20 µg/ml. For peptide epitope mapping of BPSL2096, BPSL2697 and Hsp60, synthetic peptides of 20 amino acids in length and overlapping by 10 amino acids were synthesized (GL Biochem Ltd, Shanghai) and used individually at a final concentration of 25 µg/ml (Supplementary Tables 3–5). Whole heat-inactivated Bp (HIA-Bp) was prepared from two Thai patient isolates 199a and 207a as previously described^9^ and used at a dilution of 1:60 (20 µg/ml by Bicinchoninic Acid Assay, Sigma) in ELIspot assays. The CEF T-cell epitope peptide pool (Mabtech, AB, Sweden) was used at a final concentration of 1 µg/mL.

### HLA transgenic mouse studies

This study used HLA class II transgenic mouse lines for the alleles HLA-DR1 (DRB1 * 0101) which were all maintained in the context of a homozygous knockout for murine H2-Aβ, as described previously^12–14,43–45^. Mice were maintained in individually ventilated cages and used in experiments as young adults. For T cell epitope mapping studies, mice were primed sub-cutaneously in one hind footpad with 25 μg of recombinant protein emulsified in Hunters Titermax Gold adjuvant (Sigma-Aldrich). Ten days post immunisation, the draining popliteal lymph node was removed and disaggregated into a single-cell suspension for ELISpot assays. The frequency of cells producing IFNγ in response to antigen was quantified by ELISpot (Diaclone; 2B Scientific, Oxon, U.K). Briefly, 2 × 10^5^ cells plus 25 μg/ml of protein or peptide were added to wells in HL-1 serum free medium (Lonza, Slough, U.K), supplemented with _L_-glutamine and penicillin-streptomycin (ThermoFisher Scientific, U.K). Plates were incubated for 72 h at 37 °C with 5% CO_2_. Following assay development, spots were counted on an automated ELISpot reader (Autoimmun Diagnostika, Strasbourg, France). Response frequencies were expressed as Δ spot forming cells (SFC) per 10^6^ cells, with an epitope confirmed when the majority of immunised mice responded with a magnitude greater than the mean SFCs in the absence of any antigen + 2 SD. Mean + 2 SD background SFC for each ELISpot is indicated by a dotted line.

### Human interferon gamma (IFNγ) enzyme-linked immunosorbent spot-forming cell assay (ELIspot)

Human cellular responses to protein antigens and peptide were assessed by *ex-vivo* IFNγ ELISpot assay using cryopreserved PBMC as described previously^9^. Briefly, 96-well Multiscreen-I plates (Millipore, UK) were coated overnight with 1D1K anti-human IFNγ (Mabtech, AB, Sweden) at 4 °C. PBMCs were added in triplicate wells at 2 × 10^5^ PBMCs/well and antigens added at a final concentration of 20 µg/ml. After 18 hours, secreted IFNγ was detected (Mabtech, AB, Sweden) using a CTL ELIspot reader. Results are expressed as IFNγ spot-forming cells (SFC) per million PBMC. Background (media only) responses in unstimulated control wells were typically less than 20 spots, and were subtracted from those measured in antigen-stimulated wells. Responses were considered positive if they were above the mean background response plus 2 standard deviations and above 20 SFC/10^6^ PBMC. PHA (phytohaemagglutinin) was used as a positive control to ensure T cell responsiveness, with a minimum of 100 SFC/10^6^ PBMC required to be used in the assay. All PBMC samples tested were randomised independently and ELISpot testing was performed and analysed by laboratory staff blinded to the patient group and survival status.

### Flow cytometry

PBMC were stimulated with protein (0.4 µg /well) or media only for 18 h. Brefeldin A (ebioscience, USA) was added at 10 µg/ml and following 4 h further incubation, staining for intracellular APC-IFNγ and for the immune cell surface markers PCP-anti-CD3, FITC-anti-CD4, APCH7-anti-CD8, PE-anti-CD56 and V450-CD14 (BD Biosciences, USA) was performed. Samples were analysed using a MACSQuant Analyzer 10 (Miltenyi Biotec, Germany) with Flowjo software (Treestar Inc, USA). Responses were quantitated for the negative control-subtracted percentage of IFNγ secreted CD4^+^ or CD8^+^ cells from the gated lymphocyte population.

### Enzyme-Linked Immunosorbent Assay (ELISA)

Three Bp antigens (BPSL2520, BPSS1525 or BPSS2096) or coating buffer alone were added to wells of MaxiSorp U-bottom 96-Well immunoplates (Thermo Scientific, Denmark) and incubated overnight at 4 °C. Immunoplates were blocked with 5% skimmed milk/PBS for 2 h at 37 °C. Sera from melioidosis patients or healthy control were diluted and added to the immunoplate in duplicate, then further incubated for 1 h. HRP-conjugated goat anti-human IgG (Sigma) was then added to the ELISA plate, and further incubated for 1 h. ELISAs were developed using TMB substrate. Results were measured using a Multiskan™ GO Microplate Spectrophotometer (Thermo Scientific, Wilmington, DE, USA) and expressed as absorbance value (OD450).

### Cytokine analysis

Levels of 23 cytokines were measured in the supernatants of PBMC stimulated for 18 hours with Bp antigens (BPSL2520, BPSS1525 or BPSS2096) or media alone, using the Milliplex MAP Human High sensitivity T-cell panel kit according to the manufacturer’s instructions.

### Transcriptomic analysis

PBMC from thirteen diabetic subjects with acute melioidosis were stimulated for 18 hours with either HIA-Bp, media alone or one of three Bp antigens (BPSL2520, BPSS1525, BPSL2096) for 18 h and RNA was extracted using Qiagen RNeasy minikits (Qiagen, Hilden, Germany) according to the manufacturer’s instructions. Biotinylated and amplified RNA was generated using Illumina TotalPrep RNA Amplification Kit followed by Thermo Scientific GeneJET RNA Cleanup and Concentration Micro Kit (both ThermoFisher Scientific, Waltham, MA, USA) according to the manufacturer’s instructions to generate a median of 4.9 µg RNA (range 1.6–12.0 µg), and hybridized to HumanHT-12 v4 Expression BeadChips (Illumina, San Diego, CA, USA). Arrays were scanned with an Illumina bead array reader confocal scanner, according to the manufacturer’s instructions. Array data processing and preliminary analysis with background subtraction was performed using Illumina BeadStudio software. Transcriptomics data have been deposited in the ArrayExpress database at EMBL-EBI (www.ebi.ac.uk/arrayexpress) under accession number E-MTAB-6093. 

### Data management and statistical analysis

Statistical analysis was performed using Graphpad Prism version 6 (San Diego, CA, USA) and IBM SPSS Statistics for Windows version 22.0 (Armonk, NY, USA). Non-parametric tests were used (Mann Whitney, paired Wilcoxon, Kruskal-Wallis and Spearman’s rank correlation tests) and HLA frequency comparisons were made by Chi-square, or 2-tailed Fisher’s exact test where <5 in a group. A P value (2-tailed) of <0.05 was considered significant. Transcriptomics data underwent variance stabilization and normalization in R using the vsn package from Bioconductor^46^. Further transcriptomics analysis and visual representation was performed using Partek Genomics suite with Pathway version 6.6 (St Louis, MO, USA). Differential gene expression between HIA-Bp stimulated and media alone culture conditions was performed using ANOVA paired analysis with a false discovery rate adjusted P value of <0.05 accepted as significant, with results represented graphically by volcano plots.

## Electronic supplementary material

Supplementary Information
