# Supplementary material for: Infection with Burkholderia pseudomallei – immune correlates of survival in acute melioidosis
Source: Sci Rep. 2017 Sep 22;7:12143. doi: 10.1038/s41598-017-12331-5 (PMC5610189; doi:10.1038/s41598-017-12331-5)
Supplement: Supplementary file 1 — Supplementary Information [file 41598_2017_12331_MOESM1_ESM.pdf]

Supplementary Information

Infection with *Burkholderia pseudomallei* – immune correlates of survival in acute melioidosis

Susanna J Dunachie, Kemajittra Jenjaroen, Catherine J Reynolds, Kathryn J Quigley, Ruhena Sergeant, Manutsanun Sumonwiriya, Panjaporn Chaichana, Suchintana Chumseng, Pitchayanant Ariyaprasert, Patricia Lassaux, Louise Gourlay, Charuporn Promwong, Prapit Teparrukkul, Direk Limmathurotsakul, Nicholas PJ Day, Daniel M Altmann, Rosemary J Boyton

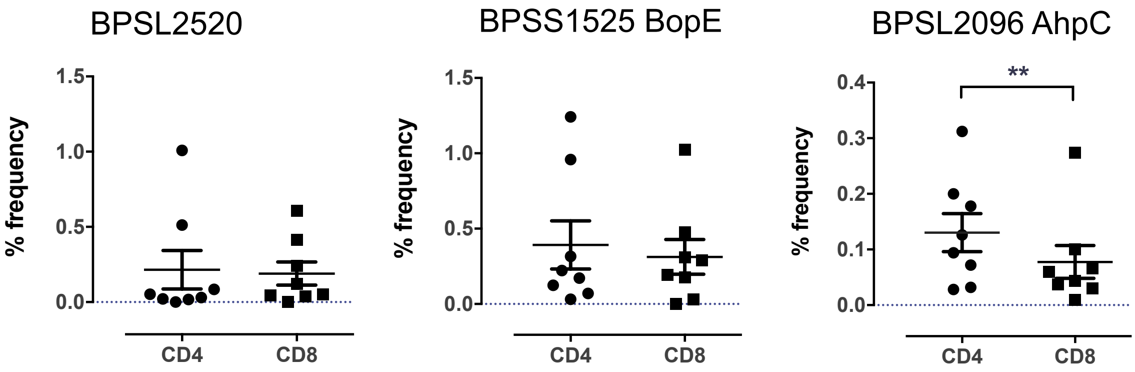

Supplementary Figure 1. T cell immunity to Bp antigen BPSL2096 is predominantly CD4 T cell mediated. (A) PBMC from 8 patients with a history of acute melioidosis 12 weeks after recovery from the acute illness were cultured in the presence of 50 μg/well of BPSL2520, BPSS1525, BPSL2096 or media alone for 18 hours before measurement of IFNγ production from $CD4^+$ (black circles) and $CD8^+$ (black squares) T cells by intracellular cytokine staining. Data are presented as % frequency of $CD4^+$ or $CD8^+$ cell populations. Error bars represent mean ± SEM and statistical significance between $CD4^+$ and $CD8^+$ populations was determined using the Mann Whitney test. **P = 0.0078

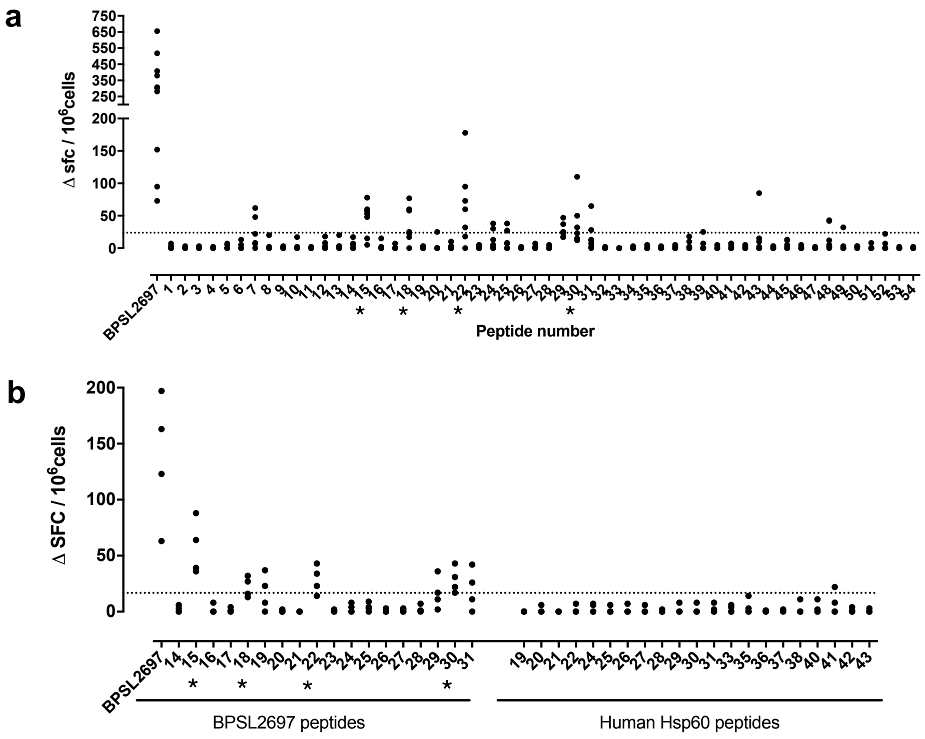

c

```
BPSL2697     1   MA------------------------AKDVVFGDSARAKMVEGVNILANAVKVTLGPKGRNVVLERSFGGPTVTKDGVSV   56
Human Hsp60  1   MLRLPTVFRQMRPVSRVLAPHLTRAYAKDVKFGADARALMLQGVDLLADAVAVTMGPKGRTVIIEQSWGSPKVTKDGVTV   80

             57  AKEIELKDKLQNMGAQMVKEVASKTSDNAGDGTTTATVLAQSIVREGMKYVASGMNPMDLKRGIDKAVAAAVEELKKISK  136
             81  AKSIDLKDKYKNIGAKLVQDVANNTNEEAGDGTTTATVLARSIAKEGFEKISKGANPVEIRRGVMLAVDAVIAELKKQSK  160
                       Peptide 15                      Peptide 18
             137 PCTTNKEIAQVGAISANSDSSIGDRIAEAMDKVGKEGVITVEDGKSLADELDVVEGMQFDRGYLSPYFINNPDKQVAVLE  216
             161 PVTTPEEIAQVATISANGDKEIGNIISDAMKKVGRKGVITVKDGKTLNDELEIIEGMKFDRGYISPYFINTSKGQKCEFQ  240
                  Peptide 22                                                     Peptide 29/30
             217 NPFVLLHDKKVSNIRDLLPVLEQVAKAGRPLLIIAEDVEGEALATLVVNNIRGILKTVAVKAPGFGDRRKAMLEDIAILT  296
             241 DAYVLLSEKKISSIQSIVPALEIANAHRKPLVIIAEDVDGEALSTLVLNRLKVGLQVVAVKAPGFGDNRKNQLKDMAIAT  320
                  Peptide 29/30
             297 GGQVIAEETGLTL--EKATLAELGQAKRIEVGKENTTIIDGAGEAVNIEARVKQIRTQIEEATSDYDREKLQERVAKLAG  374
             321 GGAVFGEE-GLTLNLEDVQPHDLGKVGEVIVTKDDAMLLKGKGDKAQIEKRIQEIIEQLDVTTSEYEKEKLNERLAKLSD  399

             375 GVAVIKVGAATEVEMKEKKARVEDALHATRAAVEEGIVPGGGVALIRARTAIAGLTGVNADQNAGIKIVLRAMEEPLRQI  454
             400 GVAVLKVGGTSDVEVNEKKDRVTDALNATRAAVEEGIVLGGGCALLRCIPALDSLTPANEDQKIGIEIIKRTLKIPAMTI  479

             455 VTNGGEEASVVVAAVAAGKGNYGYNAATGEYVDMVEAGVVDPTKVTRTALQNAASVAGLLLTTDAAVAELPKEDAP----  530
             480 AKNAGVEGSLIVEKIMQSSSEVGYDAMAGDFVNMVEKGIIDPTKVVRTALLDAAGVASLLTTAEVVVTEIPKEEKDPGMG  559

             531 MPGGMPGGMGGMGMDM  546
             560 AMGGMGGGMGG-GMF-  573
```

Supplementary Figure 2. T cell responses to Bp antigen BPSL2697 do not cross react with the human homolog Hsp60. Human HLA-DR1 transgenic mice were footpad primed with BPSL2697 as an emulsion with titermax adjuvant. 10 days post immunisation popliteal draining lymph nodes were harvested and IFNγ T cell responses to a BPSL2697 20mer peptide panel measured by ELISpot. **(a)**. A second cohort of HLA-DR1 mice were footpad primed with BPSL2697 and IFNγ T cell responses determined to the epitope rich region of the BPSL2697 peptide panel as well as the corresponding homologous region of the human Hsp60 protein. Responses were defined as positive (*) if greater than 2SD above the mean of the media only control, which is marked as a dotted line **(b)**. Homology between the Bp protein BPSL2697 and human Hsp60 is shown **(c)**.

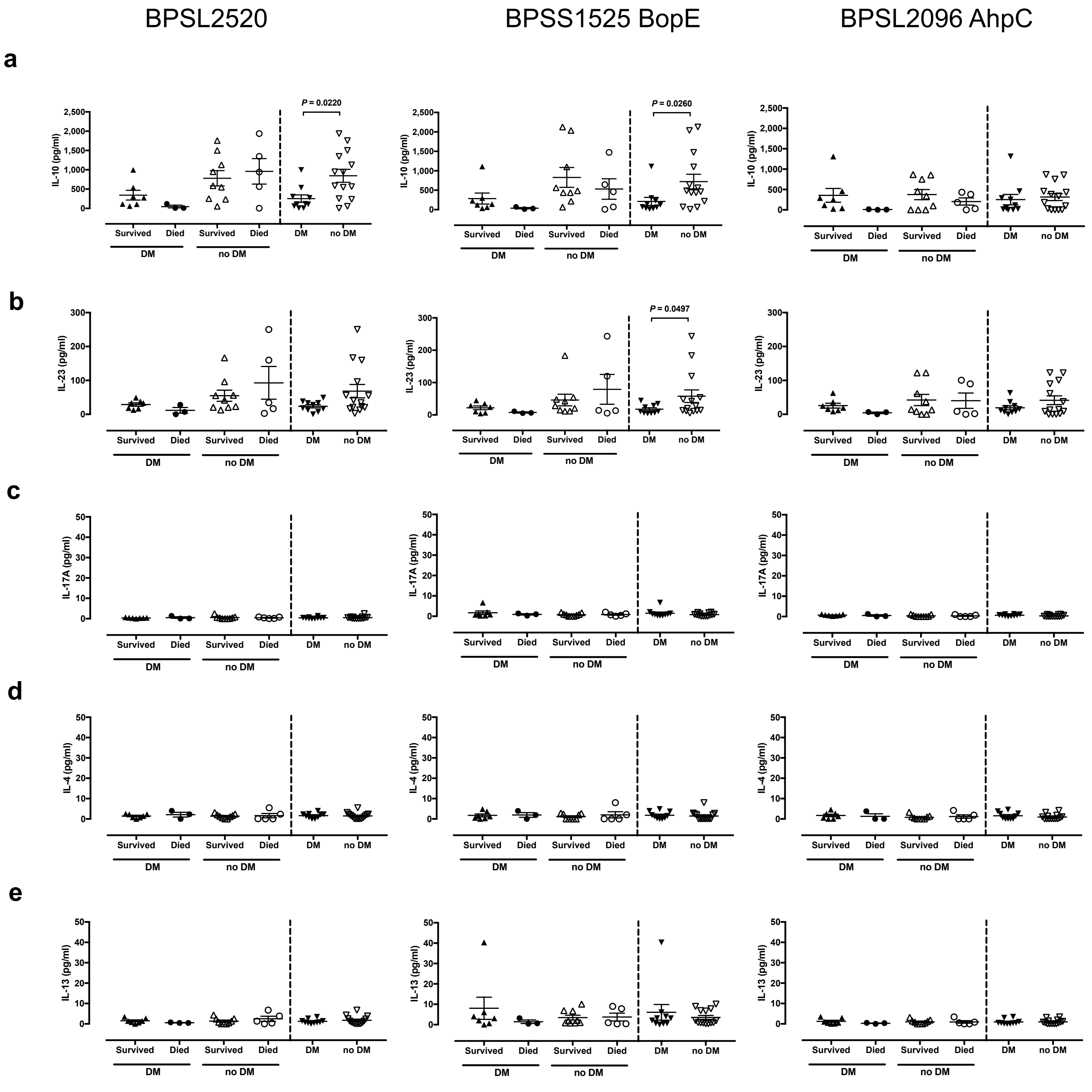

Supplementary Figure 3. Individuals with a diagnosis of diabetes have reduced IL-10 and IL-23 production in response to Bp antigen stimulation than non-diabetics. Supernatants from IFNγ ELISpot cultures using Bp antigens BPSL2520, BPSS1525 and BPSL2096 were assayed for 23 cytokines using a Milliplex MAP Human High sensitivity T cell panel kit. Data for (A) IL-10, (B) IL-23, (C) IL-17A, (D) IL-4 and (E) IL-13 are shown. Data represent 7 diabetic survivors (black triangles), 3 diabetic fatal cases (black circles), 9 non-diabetic survivors (open triangles) and 5 non-diabetic fatal cases (open circles). Error bars represent mean ± SEM and statistical significance between groups was determined using the Mann Whitney test.

S1 Table. Patient cohort demographic data

Experiment	Cohort	N	Gender		Age	Diabetes status	Figure ref
			Male n (%)	Female n (%)	Year mean (range)	n (%)	
1. HLA typing	Acute Melioidosis, survived	139	93 (67%)	46 (33%)	54 (19-89)	96 (69%)	S2 Table
	Acute melioidosis, died	44	26 (59%)	18 (41%)	59 (33-84)	27 (61%)	
2. T cell responses and survival following acute melioidosis	Acute Melioidosis, survived	31	21 (68%)	4 (31%)	55 (33-78)	6 (46%)	1, 3, 4A, 4B
	Acute melioidosis, died	13	9 (69%)	4 (31%)	54 (30-79)	16 (52%)	
3. T cell responses in survivors 12 weeks post melioidosis versus healthy seronegative controls	Survivors 12 weeks post melioidosis	31	21 (68%)	10 (32%)	54 (32-79)	17 (55%)	1, 3, 4A, 4B
	Healthy seronegative controls	21	11 (52%)	10 (48%)	43 (33-55)	0 (0%)	
4. Antibody levels	Healthy seronegative controls	21	11 (52%)	10 (48%)	43 (33-55)	0 (0%)	5
	Melioidosis (acute & 12 weeks), survived	83	60 (72%)	23 (28%)	53 (32-79)	55 (66%)	
	Acute melioidosis, died	47	28 (60%)	19 (40%)	60 (33-84)	28 (60%)	
5. T cell responses and diabetes status	Diabetic seronegative controls	20	11 (55%)	9 (45%)	56 (23-76)	20 (100%)	6
	Acute Melioidosis, diabetic	22	12 (55%)	10 (45%)	53 (32-79)	22 (100%)	
	Acute melioidosis, non-diabetic	22	18 (82%)	4 (18%)	58 (32-79)	0 (0%)	
6. Transcriptome during acute melioidosis	Diabetic acute melioidosis, survived	8	5 (63%)	3 (37%)	56 (41-67)	8 (100%)	7
	Diabetic acute melioidosis, died	5	3 (60%)	2 (40%)	53 (40-67)	5 (100%)	
7. T cell phenotyping and epitope mapping	Survivors 12 weeks post melioidosis	26	20 (77%)	6 (23%)	54 (33-73)	21 (81%)	4C, S Fig1
8. Cytokine profiling	Diabetic acute melioidosis, survived	7	4 (57%)	3 (43%)	57 (44-79)	7 (100%)	S Fig3
	Diabetic acute melioidosis, died	3	1 (33%)	2 (67%)	51 (49-63)	3 (100%)	
	Non-diabetic acute melioidosis, survived	9	6 (67%)	3 (33%)	58 (32-78)	0 (0%)	
	Non-diabetic acute melioidosis, died	5	4 (80%)	1 (20%)	61 (48-75)	0 (0%)	

S2 Table. The presence of HLA-B*46, and HLA-C*01 is associated with increased mortality following acute melioidosis.

	Survived (N=139) n (%)	Died (N=44) n (%)	P-value	Odds Ratio	95% CI
**HLA-A**					
A*1	3 (2)	0 (0)	1	NA	NA
A*2	61 (44)	21 (47)	0.7	1.2	0.6-2.3
A*3	1 (1)	1 (2)	0.4	3.2	0.2-52
A*11	78 (56)	24 (55)	0.9	0.9	0.5-1.9
A*24	41 (29)	11 (25)	0.6	0.8	0.4-1.7
A*26	3 (2)	1 (2)	1	1.1	0.1-10.4
A*29	0 (0)	1 (2)	0.2	NA	NA
A*30	10 (7)	4 (9)	0.7	1.3	0.4-4.3
A*32	2 (1)	0 (0)	1	NA	NA
A*33	31 (22)	9 (20)	0.8	0.9	0.4-2.1
A*34	7 (5)	3 (7)	0.7	1.4	0.3-5.6
A*68	3 (2)	1 (2)	1	1.1	0.1-10.4
A*74	5 (4)	0 (0)	0.3	NA	NA
**HLA-B**					
B*7	14 (10)	4 (9)	1	0.9	0.3-2.9
B*8	5 (4)	0 (0)	0.3	NA	NA
B*13	34 (24)	13 (30)	0.5	1.3	0.6-2.8
B*15	4 (3)	0 (0)	0.6	NA	NA
B*18	23 (17)	4 (9)	0.3	0.5	0.2-1.5
B*27	12 (9)	2 (5)	0.5	0.5	0.1-2.3
B*35	10 (7)	2 (5)	0.7	0.6	0.1-2.9
B*38	12 (9)	3 (7)	1	0.8	0.2-2.9
B*39	13 (9)	3 (7)	0.8	0.7	0.2-2.6
B*40	1 (1)	0 (0)	1	NA	NA
B*44	9 (6)	4 (9)	0.5	1.4	0.4-4.9
**B*46**	**30 (22)**	**19 (43)**	**0.005**	**2.8**	**1.3-5.7**

B*47	1 (1)	0 (0)	1	NA	NA
B*48	0 (0)	1 (2)	0.2	NA	NA
B*51	15 (11)	3 (7)	0.6	0.6	0.2-2.2
B*52	3 (2)	1 (2)	1	1.1	0.1-10.4
B*54	0 (0)	1 (2)	0.2	NA	NA
B*55	3 (2)	0 (0)	1	NA	NA
B*56	6 (4)	3 (7)	0.5	1.6	0.4-6.8
B*57	0 (0)	1 (2)	0.2	NA	NA
B*58	22 (17)	5 (11)	0.5	0.7	0.2-1.9
B*60	9 (6)	3 (7)	1	1.1	0.3-4.1
B*61	3 (2)	1 (2)	1	1.1	0.1-10.4
B*62	18 (13)	10 (23)	0.1	2.0	0.8-4.7
B*69	1 (1)	0 (0)	1	NA	NA
B*75	14 (10)	1 (2)	0.1	0.2	0.0-1.6
B*76	1 (1)	0 (0)	1	NA	NA
**HLA-C**					
**C*1**	**32 (23)**	**21 (48)**	**0.002**	**3.1**	**1.5-6.2**
C*3	2 (1)	0 (0)	1	NA	NA
C*4	39 (28)	7 (16)	0.1	0.5	0.2-1.2
C*6	9 (6)	4 (9)	0.5	1.4	0.4-4.9
C*7	68 (49)	25 (57)	0.4	1.4	0.7-2.7
C*8	16 (12)	2 (5)	0.3	0.4	0.1-1.7
C*9	5 (4)	0 (0)	0.3	NA	NA
C*10	48 (35)	15 (34)	1	1.0	0.5-2.0
C*12	8 (6)	2 (5)	1	0.8	0.2-3.8
C*14	9 (6)	1 (2)	0.5	0.3	0.0-2.7
C*15	10 (9)	4 (7)	0.7	1.3	0.4-4.3

The number of individuals (n, %) carrying at least one copy of the allele is shown. Allele and genotype frequency was determined by direct counting. The odds ratio (OR) and 95% confidence interval (CI) were calculated. Allele and genotype frequency comparisons were made by Chi-square or Fisher's exact test (where <5 in a group). P values less than or equal to 0.005 were regarded as significant and are displayed in boldface. NS = not significant; NA = not applicable.

S3 Table. Protein antigen peptide panels (10aa overlap) for *Burkholderia pseudomallei*, BPSL2096 (AhpC)

Peptide number	Sequence
1	MKTVGDKLEAFTVVAAKPGF
2	FTVVAAKPGFNNHEENGQSA
3	NNHEENGQSAFETVTEASFP
4	FETVTEASFPGKWKIIYFYP
5	GKWKIIYFYPKDFTFVCPTE
6	KDFTFVCPTEIVEFAKLAKQ
7	IVEFAKLAKQFEERDAVLLG
8	FEERDAVLLGGSSDNEFVKL
9	GSSDNEFVKLAWRREHKDLD
10	AWRREHKDLDKLNHYSFGDV
11	KLNHYSFGDVKGELIDQLGV
12	KGELIDQLGVRDKEAGVALR
13	RDKEAGVALRATFIVDPDNT
14	ATFIVDPDNTIQHVSVNNLN
15	IQHVSVNNLNVGRSPEEILR
16	VGRSPEEILRILDGLQTDEL
17	ILDGLQTDELCPCNRAIGGATL

S4 Table. Protein antigen peptide panels (10aa overlap) for *Burkholderia pseudomallei*, BPSL2697 (GroEL)

Peptide number	Peptide sequence
1	MAAKDVVFGDSARAKMVEGV
2	SARAKMVEGVNILANAVKVT
3	NILANAVKVTLGPKGRNVVL
4	LGPKGRNVVLERSFGGPTVT
5	ERSFGGPTVTKDGVSVAKEI
6	KDGVSVAKEIELKDKLQNMG
7	ELKDKLQNMGAQMVKEVASK
8	AQMVKEVASKTSDNAGDGTT
9	TSDNAGDGTTTATVLAQSIV
10	TATVLAQSIVREGMKYVASG
11	REGMKYVASGMNPMDLKRGI
12	MNPMDLKRGIDKAVAAAVEE
13	DKAVAAAVEELKKISKPCTT
14	LKKISKPCTTNKEIAQVGAI
15	NKEIAQVGAISANSDSSIGD
16	SANSDSSIGDRIAEAMDKVG
17	RIAEAMDKVGKEGVITVEDG
18	KEGVITVEDGKSLADELDVV
19	KSLADELDVVEGMQFDRGYL
20	EGMQFDRGYLSPYFINNPDK
21	SPYFINNPDKQVAVLENPFV
22	QVAVLENPFVLLHDKKVSNI
23	LLHDKKVSNIRDLLPVLEQV
24	RDLLPVLEQVAKAGRPLLII
25	AKAGRPLLIIAEDVEGEALA
26	AEDVEGEALATLVVNNIRGI
27	TLVVNNIRGILKTVAVKAPG
28	LKTVAVKAPGFGDRRKAMLE
29	FGDRRKAMLEDIAILTGGQV
30	DIAILTGGQVIAEETGLTLE
31	IAEETGLTLEKATLAELGQA
32	KATLAELGQAKRIEVGKENT
33	KRIEVGKENTTIIDGAGEAV
34	TIIDGAGEAVNIEARVKQIR
35	NIEARVKQIRTQIEEATSDY
36	TQIEEATSDYDREKLQERVA
37	DREKLQERVAKLAGGVAVIK
38	KLAGGVAVIKVGAATEVEMK
39	VGAATEVEMKEKKARVEDAL
40	EKKARVEDALHATRAAVEEG
41	HATRAAVEEGIVPGGGVALI
42	IVPGGGVALIRARTAIAGLT
43	RARTAIAGLTGVNADQNAGI
44	GVNADQNAGIKIVLRAMEEP
45	KIVLRAMEEPLRQIVTNGGE
46	LRQIVTNGGEEASVVVAAVA
47	EASVVVAAVAAGKGNYGYNA
48	AGKGNYGYNAATGEYVDMVE
49	ATGEYVDMVEAGVVDPTKVT
50	AGVVDPTKVTRTALQNAASV
51	RTALQNAASVAGLLLTTDAA
52	AGLLLTTDAAVAELPKEDAP
53	VAELPKEDAPMPGGMPGGMG
54	MPGGMPGGMGGMGMDM

S5 Table. Protein antigen peptide panels (10aa overlap) for HSP60

Peptide number	Peptide sequence	Peptide number	Peptide sequence
1	MLRLPTVFRQMRPVSR	37	NRLKVGLQVVAVKAPG
2	RQMRPVSRVLAPHLTR	38	VVAVKAPGFGDNRKNQ
3	VLAPHLTRAYAKDVKF	39	FGDNRKNQLKDMAIAT
4	AYAKDVKFGADARALM	40	LKDMAIATGGAVFGEE
5	GADARALMLQGVDLLA	41	GGAVFGEEGLTLNLED
6	LQGVDLLADAVAVTMG	42	GLTLNLEDVQPHDLGK
7	DAVAVTMGPKGRTVII	43	VQPHDLGKVGEVIVTK
8	PKGRTVIIEQSWGSPK	44	VGEVIVTKDDAMLLKG
9	EQSWGSPKVTKDGVTV	45	DDAMLLKGKGDKAQIE
10	VTKDGVTVAKSIDLKD	46	KGDKAQIEKRIQEIIE
11	AKSIDLKDKYKNIGAK	47	KRIQEIIEQLDVTTSE
12	KYKNIGAKLVQDVANN	48	QLDVTTSEYEKEKLNE
13	LVQDVANNTNEEAGDG	49	YEKEKLNERLAKLSDG
14	TNEEAGDGTTTATVLA	50	RLAKLSDGVAVLKVGG
15	TTTATVLARSIAKEGF	51	VAVLKVGGTSDVEVNE
16	RSIAKEGFEKISKGAN	52	TSDVEVNEKKDRVTDA
17	EKISKGANPVEIRRGV	53	KKDRVTDALNATRAAV
18	PVEIRRGVMLAVDAVI	54	LNATRAAVEEGIVLGG
19	MLAVDAVIAELKKQSK	55	EEGIVLGGGCALLRCI
20	AELKKQSKPVTTPEEI	56	GCALLRCIPALDSLTP
21	PVTTPEEIAQVATISA	57	PALDSLTPANEDQKIG
22	AQVATISANGDKEIGN	58	ANEDQKIGIEIIKRTL
23	NGDKEIGNIISDAMKK	59	IEIIKRTLKIPAMTIA
24	IISDAMKKVGRKGVIT	60	KIPAMTIAKNAGVEGS
25	VGRKGVITVKDGKTLN	61	KNAGVEGSLIVEKIMQ
26	VKDGKTLNDELEIIEG	62	LIVEKIMQSSSEVGYD
27	DELEIIEGMKFDRGYI	63	SSSEVGYDAMAGDFVN
28	MKFDRGYISPYFINTS	64	AMAGDFVNMVEKGIID
29	SPYFINTSKGQKCEFQ	65	MVEKGIIDPTKVVRTA
30	KGQKCEFQDAYVLLSE	66	PTKVVRTALLDAAGVA
31	DAYVLLSEKKISSIQS	67	LLDAAGVASLLTTAEV
32	KKISSIQSIVPALEIA	68	SLLTTAEVVVTEIPKE
33	IVPALEIANAHRKPLV	69	VVTEIPKEEKDPGMGA
34	NAHRKPLVIIAEDVDG	70	EKDPGMGAMGGMGGGM
35	IIAEDVDGEALSTLVL	71	MGGMGGGMGGGMF
36	EALSTLVLNRLKVGLQ		

S6 Table. Patient cohort comorbidity data

Presence or absence of diabetes<br>Number of patients (n)	Diabetes<br>123		No diabetes<br>60		*P* value<br>(Fisher)
Mean age (range, SD)	53 (19-89, 12.1)		58 (25-88, 14.4)		**0.056***
1 or more non-DM comorbidity	45	37%	32	53%	**0.038**
2 or more non-DM comorbidity	11	9%	16	27%	**0.003**
3 or more non-DM comorbidity	1	1%	3	5%	0.1
Age ≥ 65 yrs	19	15%	17	28%	**0.048**
Renal disease	20	16%	13	22%	0.41
Alcohol excess	3	2%	7	12%	**0.015**
IHD	14	11%	9	15%	0.64
Lung disease	0	0%	1	2%	-
Liver disease	2	2%	5	8%	**0.039**

* Age comparison by Mann-Witney

S7 Table: Top 100 genes upregulated in human PBMCs upon stimulation with Bp

Gene name	Fold-Change	P value
PTGS2	17.76	8.58E-15
IL6	16.02	3.58E-13
CSF2	12.50	8.60E-17
CCL20	7.90	7.85E-12
IL1A	7.06	1.87E-10
TNFAIP6	6.21	1.15E-12
IL1F9	6.05	1.01E-10
IDO1	6.00	3.18E-12
EBI3	5.91	3.91E-14
CCL3L1	5.80	3.29E-15
INDO	5.79	5.36E-12
IL24	5.73	5.06E-10
CXCL2	5.64	2.35E-13
CCL3	5.59	8.29E-15
SERPINB7	4.54	2.66E-09
IL1B	4.49	5.12E-06
IL19	4.41	1.64E-07
IL23A	3.66	2.33E-10
CCL3L3	3.59	1.22E-11
F3	3.47	6.40E-10
MIR155HG	3.32	8.54E-17
RIPK2	3.30	2.20E-12
SOD2	3.25	1.30E-12
CXCL1	3.23	1.02E-06
TFPI2	3.09	3.51E-10
NAMPT	2.96	2.11E-08
MMP1	2.92	8.02E-06
GJB2	2.88	9.56E-10
LOC654103	2.80	1.75E-08
OSM	2.78	3.87E-08
MMP10	2.77	1.32E-08
SLC25A37	2.74	3.00E-08
IRAK2	2.72	1.16E-10
C1QTNF1	2.63	1.68E-07
IL2RA	2.60	1.23E-08
TM4SF1	2.57	8.18E-07
ADORA2A	2.46	8.99E-09
IER3	2.46	2.10E-09
PFKFB3	2.43	2.96E-10
LOC387763	2.42	1.84E-09
LOC653778	2.35	8.23E-08
CKB	2.30	4.82E-12

DDIT4	2.30	2.91E-11
MAP1LC3A	2.29	1.02E-06
DUSP5	2.29	5.23E-10
SERPINA1	2.14	5.68E-06
IL8	2.13	3.58E-05
MARCKS	2.12	8.31E-09
BMP6	2.11	2.69E-09
GRAMD1A	2.09	1.47E-06
MAP3K8	2.08	2.95E-10
SERPINB2	2.07	2.58E-06
KIAA1199	2.05	0.000587364
PANX2	2.01	4.44E-10
LAD1	2.01	1.11E-05
LOC143666	2.00	3.16E-10
OSGIN2	1.99	5.22E-10
SYTL3	1.95	3.61E-14
VNN3	1.95	1.94E-06
NFKBIZ	1.95	3.24E-09
ADA	1.94	4.92E-07
MPZL1	1.94	7.19E-10
CCL4L2	1.93	3.12E-07
PDE4B	1.93	1.83E-09
TNFRSF4	1.93	9.29E-10
LAG3	1.89	4.51E-09
CXCL6	1.88	4.10E-05
NFKB2	1.88	1.87E-12
TRAF1	1.87	6.73E-10
RNF144B	1.87	4.94E-08
GBP4	1.87	6.31E-09
BASP1	1.85	1.05E-09
SLC25A24	1.85	4.06E-06
D2HGDH	1.84	2.16E-09
DLL1	1.83	2.22E-06
FAM108C1	1.82	2.92E-06
PALLD	1.81	2.42E-08
GBP5	1.81	1.13E-08
LOC728835	1.79	3.73E-06
CD7	1.79	2.34E-12
PMAIP1	1.78	2.48E-10
SGPP2	1.78	1.93E-07
ZC3H12A	1.76	1.46E-08
NFKBIA	1.74	7.96E-11
SLA2	1.74	3.25E-12
LOC728830	1.73	8.61E-05
CYP4B1	1.73	1.50E-07
EBF1	1.73	2.25E-08

DENND5A	1.72	9.50E-09
SOCS1	1.72	2.04E-08
ITGB8	1.71	1.28E-08
CD83	1.69	6.00E-10
TNFAIP3	1.68	1.36E-10
CXCR4	1.67	2.91E-09
SOCS3	1.66	6.98E-05
USP36	1.65	2.50E-11
IL15RA	1.65	6.16E-12
ITGA1	1.64	1.32E-08
DUSP1	1.64	3.09E-05
IL10	1.64	1.51E-05

S8 Table: Top 100 genes downregulated in human PBMCs upon stimulation with Bp

Gene name	Fold-Change	P value
CSF1R	-9.68	5.33E-20
TGFBI	-9.08	8.87E-13
MMP9	-6.73	4.14E-15
FPR3	-6.32	1.54E-14
GPNMB	-6.25	1.65E-12
RNASE1	-5.72	9.57E-10
FUCA1	-5.34	2.84E-11
CYP27A1	-5.29	6.34E-18
SPRED1	-5.22	3.49E-18
FCN1	-4.94	9.70E-14
CYBB	-4.83	8.87E-15
OLR1	-4.57	1.38E-09
FBP1	-4.53	1.00E-11
TIMP2	-4.48	7.45E-16
CCR1	-4.37	2.69E-13
SLAMF8	-4.28	7.31E-18
SLCO2B1	-4.28	4.59E-12
LYZ	-4.27	1.91E-09
AIF1	-4.27	4.18E-15
TNS3	-4.23	6.85E-18
CD9	-4.04	2.19E-12
CD163	-4.01	9.06E-11
CLEC4A	-3.86	3.09E-12
IFI30	-3.81	2.24E-15
SPP1	-3.78	9.68E-12
CTSB	-3.68	4.19E-16
FLJ22662	-3.63	2.36E-12
CCL7	-3.49	2.39E-07
IL18BP	-3.42	5.16E-12
CST3	-3.41	4.54E-15
C4ORF18	-3.40	1.52E-11
EGR2	-3.31	5.18E-12
KCTD12	-3.30	2.51E-12
CD36	-3.26	4.85E-10
GSN	-3.24	9.81E-15
FCGRT	-3.23	9.35E-16
MT1H	-3.20	9.00E-08
CEBPA	-3.15	3.79E-16
ACP5	-3.14	1.69E-12
ANXA2P1	-3.12	4.94E-14
C5AR1	-3.12	2.95E-16
FAM20C	-3.11	8.02E-15

RAB7B	-3.09	5.28E-15
MERTK	-3.01	1.43E-09
CLEC5A	-2.98	3.55E-09
NPL	-2.97	1.46E-13
DAB2	-2.97	1.60E-16
CAMK1	-2.96	3.17E-13
SLC38A6	-2.90	2.84E-17
IGSF6	-2.90	6.86E-13
RGL1	-2.89	1.61E-09
DUSP6	-2.88	1.68E-15
C19ORF59	-2.86	9.36E-05
LHFPL2	-2.84	2.54E-11
SLC11A1	-2.81	6.76E-08
FLVCR2	-2.79	1.10E-14
AADACL1	-2.76	4.93E-14
RNASE6	-2.75	1.41E-12
SCARB2	-2.74	2.62E-14
ADAP2	-2.74	3.97E-11
GPR162	-2.70	2.11E-15
SIRPA	-2.68	1.20E-17
SGK1	-2.65	1.97E-12
STAB1	-2.64	2.88E-12
CCL2	-2.63	4.57E-06
HK3	-2.62	9.33E-13
ABCC3	-2.62	4.60E-14
FOS	-2.61	7.57E-12
CCL24	-2.61	9.72E-06
SGK	-2.59	2.03E-12
LIPA	-2.59	2.26E-08
PMP22	-2.57	5.15E-07
CXCL16	-2.56	5.53E-07
DPYSL2	-2.55	9.10E-16
PLD3	-2.55	8.47E-12
AVPI1	-2.54	5.87E-13
PLA2G7	-2.50	1.66E-09
MT1G	-2.50	3.50E-06
TMEM51	-2.50	1.31E-11
LMNA	-2.46	6.38E-12
ITGAM	-2.45	3.35E-14
ENG	-2.45	1.90E-19
EMP1	-2.42	2.53E-07
ANXA2	-2.42	7.99E-17
TYROBP	-2.39	4.09E-13
EMR2	-2.38	1.27E-09
PAPSS1	-2.38	5.78E-16
MAFB	-2.37	2.10E-11

CD14	-2.36	3.01E-05
FER1L3	-2.36	3.30E-12
DHRS9	-2.36	1.17E-07
SCG5	-2.36	4.06E-06
CD68	-2.35	3.04E-11
FGL2	-2.34	2.30E-09
LILRB2	-2.32	8.32E-10
PDK4	-2.30	9.45E-10
CD276	-2.29	1.96E-17
CLEC12A	-2.27	5.31E-08
SORT1	-2.25	7.85E-13
CX3CR1	-2.23	7.46E-11
